# Associations between epigenetic and inflammatory aging, cognitive function, and blood‐based biomarkers of Alzheimer’s disease: findings from the INSPIRE‐T cohort

**DOI:** 10.1002/alz70861_108934

**Published:** 2025-12-23

**Authors:** Samuel Thuriot, Jason Shourick, David Furman, Jean Marc Lemaitre, Sophie Guyonnet, Bruno Vellas, Sandrine Andrieu, Laure Rouch

**Affiliations:** ^1^ IHU HealthAge CERPOP UMR 1295, Toulouse France; ^2^ IHU HealthAge CERPOP UMR 1205, Toulouse France; ^3^ Buck Institute for Research on Aging, Novato, CA USA; ^4^ IRMB, Montpellier France; ^5^ IHU HealthAge INSERM 1295 CERPOP, Toulouse France

## Abstract

**Background:**

DNA methylation and systemic age‐related inflammation patterns have been employed to develop epigenetic and inflammatory aging clocks as a measure of biological aging. The extent to which it impacts cognition remains unclear, and the link with blood‐based biomarkers of Alzheimer’s disease (AD) has yet to be investigated.

**Methods:**

We included 586 participants (median age 51 [37‐61] years, women 66%) from the INSPIRE‐T cohort. At baseline, 5 measures of epigenetic age accelerations were considered from Horvath pan tissue, Horvath skin and blood, Hannum, PhenoAge and GrimAge epigenetic aging clocks. Inflammatory age acceleration was estimated from iAge clock. Biological age accelerations were calculated as the residuals from a linear regression of biological age on chronological age. Cognition was assessed at baseline and annually over 3 years. Global cognition was calculated as a composite measure using the average of Z scores for the MMSE orientation items (orientation), FCSRT (episodic memory), DSST (processing speed) and CNT (verbal fluency) tests. Each domain was also assessed separately. Blood NFL and *p* ‐tau217 were measured at baseline.

**Results:**

In cross‐sectional analyses and after adjustment for chronological age and sex, only GrimAge accelerated epigenetic aging was associated with lower global cognition (β = ‐0.18, 95% CI [‐0.33; ‐0.03], *p* =0.02). Further adjustment for education and ApoE4 genotype did not modify the results. Accelerated inflammatory aging was not associated with global cognition (*p* =0.55). Longitudinal analyses did not reveal any significant interaction effect between GrimAge epigenetic aging and advancing chronological age. Skin and blood Horvath’s accelerated epigenetic aging was associated with lower processing speed (β = ‐ 1.49, 95% CI [‐2.70; ‐0.27], *p* =0.02) with advancing age. In fully adjusted models, neither epigenetic nor inflammatory accelerated aging were significantly associated with *p* ‐tau217 levels (*p* >0.05 for all clocks). However, accelerated inflammatory aging was associated with higher NFL levels (β = 0.76, 95% CI [0.23; 1.30], *p* <0.01).

**Conclusions:**

Our findings showed that accelerated GrimAge epigenetic aging was associated with lower cognition. Accelerated inflammatory aging was linked to higher blood NFL levels. Our research could pave the way for an early molecular identification of at‐risk individuals, enabling personalized medicine interventions to promote brain health.

